# Brain Tumours: Rise in Glioblastoma Multiforme Incidence in England 1995–2015 Suggests an Adverse Environmental or Lifestyle Factor

**DOI:** 10.1155/2018/7910754

**Published:** 2018-06-24

**Authors:** Alasdair Philips, Denis L. Henshaw, Graham Lamburn, Michael J. O'Carroll

**Affiliations:** ^1^Children with Cancer UK, 51 Great Ormond Street, London, WC1N 3JQ, UK; ^2^Powerwatch, Cambridgeshire, UK; ^3^Professor Emeritus, University of Bristol, UK; ^4^Professor Emeritus, Vice–Chancellor's Office, University of Sunderland, UK

## Abstract

**Objective:**

To investigate detailed trends in malignant brain tumour incidence over a recent time period.

**Methods:**

UK Office of National Statistics (ONS) data covering 81,135 ICD10 C71 brain tumours diagnosed in England (1995–2015) were used to calculate incidence rates (ASR) per 100k person–years, age–standardised to the European Standard Population (ESP–2013).

**Results:**

We report a sustained and highly statistically significant ASR rise in glioblastoma multiforme (GBM) across all ages. The ASR for GBM more than doubled from 2.4 to 5.0, with annual case numbers rising from 983 to 2531. Overall, this rise is mostly hidden in the overall data by a reduced incidence of lower-grade tumours.

**Conclusions:**

The rise is of importance for clinical resources and brain tumour aetiology. The rise cannot be fully accounted for by promotion of lower–grade tumours, random chance or improvement in diagnostic techniques as it affects specific areas of the brain and only one type of brain tumour. Despite the large variation in case numbers by age, the percentage rise is similar across the age groups, which suggests widespread environmental or lifestyle factors may be responsible. This article reports incidence data trends and does not provide additional evidence for the role of any particular risk factor.

## 1. Introduction

The causes of brain tumours in adults remain largely unknown [1]. In 2011, the World Health Organisation (WHO) prioritised the monitoring of detailed brain tumour incidence trends through population–based cancer registries [2]. This article reports recent changes in malignant brain tumour incidence in England that include age, sex, morphology and tumour location.

## 2. Materials and Methods

### 2.1. Data

The International Classification of Diseases for Oncology (ICD–O) is a dual classification, with coding systems for both topography and morphology [3]. The relevant topology codes are listed in Table 1, along with the number of tumours diagnosed in 1995 and 2015.

There are 102 different ICD–O–3.1 morphology codes used in the data set, though many have few cases. The morphology code describes the cell type and its biological activity / tumour behaviour.

WHO last updated their classifications in 2016, but their changes have minimal impact on our analysis of the data [4, 5]. Malignant brain neoplasms without histology are recorded as ICD–10 D43 (D43.0 & D43.2 supratentorial).

We used anonymised individual–level national cancer registration case data from the UK Office of National Statistics (ONS) for all 81,135 ICD10–C71 category primary malignant brain tumours diagnosed in England for the years from 1995 to 2015, plus 8,008 ICD10–D43 supratentorial malignant tumours without histology/morphology data from 1998–2015. The initial data is supplied by the National Cancer Registration Service (NCRS). The ONS then apply further validation checks and the UK Department of Health use the ONS data to inform policy making. The ONS state their cancer data are generally within 2% of the correct values [6]. Until about 2005, some cases in the oldest age–groups will not have been recorded in the cancer registries. Since 2005 this error is likely to be small.

Glioblastoma Multiforme (GBM), the most common and most malignant primary tumour of the brain, is associated with one of the worst five–year survival rates among all human cancers, with an average survival from diagnosis of only about 1 year. This ensures that few cases will be unrecorded in the ONS database and we show that their number of GBM tumours is similar to NHS hospital inpatient numbers. The data include the year of diagnosis, age at diagnosis, sex of patient, primary site and morphology code. National population estimates of age and gender by calendar year were also obtained from ONS data [7] and age–specific incidence rates per 100,000 person–years and for a wide variety of tumour types were calculated in 5-year age group bins for males and females separately.

Some published incidence analyses have used different criteria as to which glioma and astrocytoma should be considered malignant. WHO considers Grades I to IV as biologically malignant even if they have not been graded histologically malignant. We have taken the WHO/IARC morphology behaviour codes /3, /6 and /9 as being histologically malignant which means that Grade I and II tumours are classed as low–grade malignancies.

We are not aware of any specific bias in the ONS data. There is a slight data–lag in cancer registry data, which are regularly checked and updated if necessary, but are generally stable after 3 to 5 years. Our ONS data extract is dated 4^th^ July 2017.

Brodbelt et al. (2015) [8] reported an analysis of treatment and survival for 10,743 GBM cases in England over the period 2007–2011, which had an overall median survival of only 6.1 months, rising to 14.9 months with maximal treatment. Brodbelt et.al.'s GBM case total from English hospital data is only 0.5% higher that our ONS GBM total of 10,687 cases for the same time period; this suggests that a very complete UK cancer diagnosis and registration system is now in place. In contrast, Ostrom et al. (2015) [9] reporting on USA SEER brain tumour data provide a scatter–plot that shows a median complete registration and histological confirmation level of only about 65%, with the best examples returning less than 75% full completion in 2012.

### 2.2. Confounding

We had a large number of categories and sub–categories in the data. It was necessary to combine some of these to increase the resolving power. We ran analyses separately for each site (C71.0 to C71.9), for each main type of tumour, and for tumour grade (I to IV). It was immediately obvious that the most significant change was in the incidence of GBM in frontal and temporal lobes. The obvious potential confounders would be the C71.8 (overlapping) and C71.9 (unspecified) categories due to better imaging techniques and we discuss this later.

### 2.3. Standardisation

Incidence rates rise dramatically with age and standardisation is necessary as population age profiles are changing with time. We calculated age–standardised incidence rates (ASR) per 100k person–years to the current recommended European Standard Population (ESP–2013), as it best represents the reality of the case burden on society [10]. Adjusting European cancer incidence to the World Standard Population is not helpful as the age-spectra are so different.

Table 2 lists the morphology codes with the highest case numbers, totalling 80354 tumours. Included in our analyses are an additional 781 cases in 78 other categories, each with fewer than 100 cases over the 21 years. A full listing of all the cases in the data set is provided in the Supplementary File [S1].

We needed to group data to improve resolution and reduce random data noise. We examined infant and child neoplasms separately, but did not find any statistically significant time–trends. Three age-groups seemed reasonable. We chose a child, teenage and young-adult group (0-29), a main middle-age group (30-54) and an older age group (over 55 years of age). These reasonably split the population into three roughly equal (20, 18 and 16 million) groups of people. The case totals in the three groups were about 9.5k, 19.5k and 52k respectively. We tested moving the cut-point boundaries by 5 years in both directions and it made little difference to the overall results.

### 2.4. Analysis

The cases were analysed by morphology, topology, sex, age, age–specific and age–standardised incidence. The Annual Average Percentage Change (AAPC) and corresponding 95% CI and p–values were calculated using Stata SE12.1 (StataCorp). A linear model on the log of the age–standardised rates, which tests for a constant rate of change (e^(ln(rate))^), best fitted the data. See Supplementary File sections [S2] and [S3].

### 2.5. Background

In a major 2013 review article, Hiroko Ohgaki and Paul Kleihues [11] wrote “Glioblastoma is the most frequent and malignant brain tumor. The vast majority of glioblastomas (~90%) develop rapidly* de novo* in elderly patients, without clinical or histologic evidence of a less malignant precursor lesion (primary glioblastomas). Secondary glioblastomas progress from low-grade diffuse astrocytoma or anaplastic astrocytoma. They manifest in younger patients, have a lesser degree of necrosis, are preferentially located in the frontal lobe, and carry a significantly better prognosis.”

**Figure 1:**
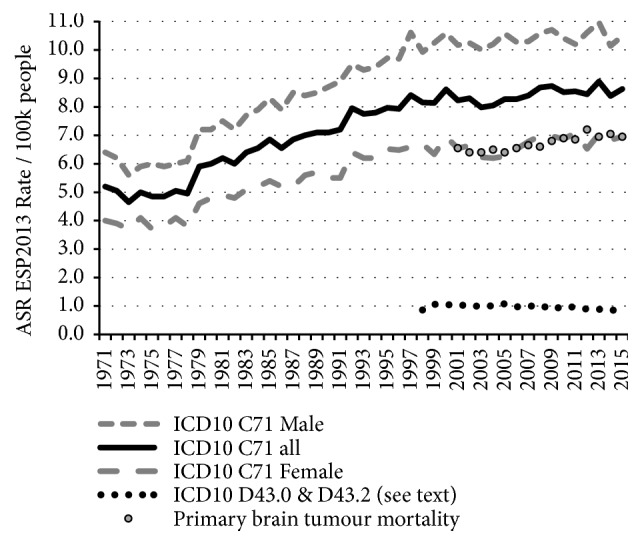
Age–standardised overall trends from 1971 to 2015 using data in ONS MB1 series, including a smaller number of supratentorial neoplasms without histology or morphology data coded D43.0 & D43.2. The data table for this figure is in the SI file as [S4].

Overall primary malignant brain tumour ASRs are only rising slowly and are often considered fairly static.  Figure 1 shows the age–standardised trends from 1971 to 2015. From the 1970s to about 2000 there was a fairly steady rise in recorded overall incidence, however since then the rise has slowed, though clinicians have been reporting a rise in high-grade, aggressive tumours.

Overall adult survival for all malignant brain tumours after diagnosis during 2006–2010 was about 35% for one year and 15% for five years, falling to about 3% for aggressive grades–III and IV tumours. ONS data show age-standardised death rates from malignant brain tumours (C71) have increased by 7% between 2001 and 2015, showing that improvements in treatment alone are inadequate and that there is a need to find ways of preventing brain cancer [12].

## 3. Results

**Figure 2:**
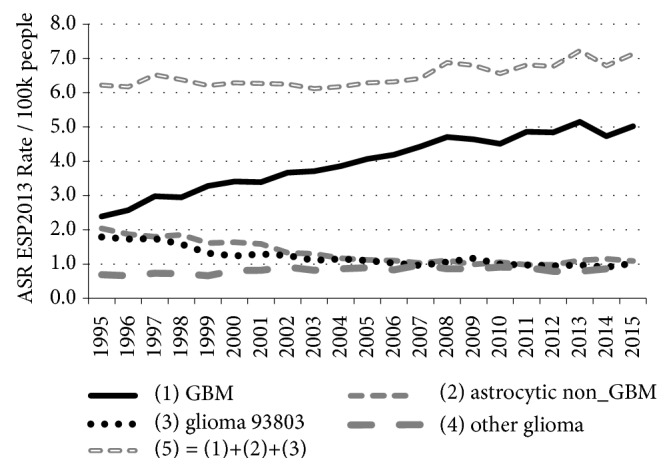
Age–standardised incidence rates for all C71 glioma cases diagnosed between 1995 and 2015 analysed by type and year (Data in Table 3). Grouping details: (1) = 94403–94433 (2) = 93843, 94003–94303 (3) = 93803 (4) = 93813, 93823, 93903–93943, 94503–94733.

Comparing new case numbers in 2015 with 1995 shows an extra 1548 aggressive GBM tumour cases annually.  Figure 2 and Table 3 show that up to about 2004 the overall rise in GBM incidence (Annual Average Percentage Change (AAPC) 5.2%, 95% CI 3.7–6.6, p < 0·00003) could be mostly compensated for by the fall in incidence of all lower grade astrocytoma and “glioma, malignant, NOS, ICD10–93803”. This leaves a fairly steady rise in the GBM ASR from 2004 to 2015 (AAPC 2.2%, 95% CI 1.4–3.0, p < 0.0001).

**Figure 3:**
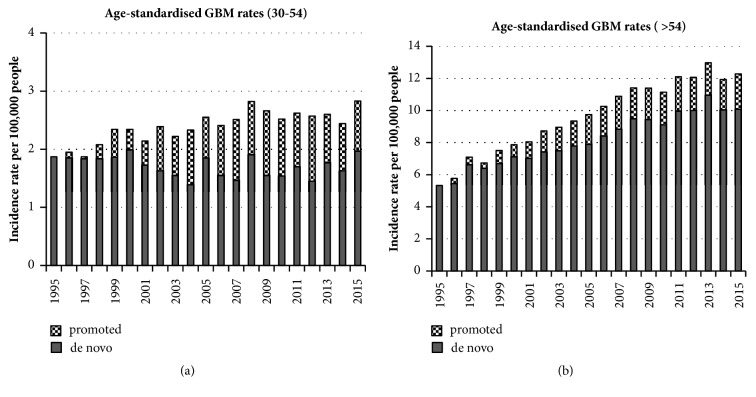
Age–standardised rates for two age groups. The possible split between* de novo* and secondary promoted GBMs is based on incidence change of Grades II and III diffuse and anaplastic astrocytoma.

Ohgaki and Kleihues [11] reported that most secondary GBMs are found in younger middle-age people and most primary GBMs are in over 60s. We tested our (30–54) and (>54) age group data, splitting the total GBM into* de novo* and promoted tumours. We estimated the maximum possible number of promoted tumours using the change in the grades II and III diffuse and anaplastic astrocytomas. The results are shown in Figures 3(a) and 3(b). These are discussed later.

We found a large decrease of ASR over time for Grade–II diffuse astrocytoma, a slight rise in ASR for WHO Grade–III anaplastic astrocytoma (94013; 2832 cases). There was little change in rates of anaplastic oligodendroglioma (94513; 1339 cases), anaplastic ependymoma (93923; 313 cases) Grade–II oligodendroglioma (94503; 2671cases), embryonal, or ependymal tumours.

**Figure 4:**
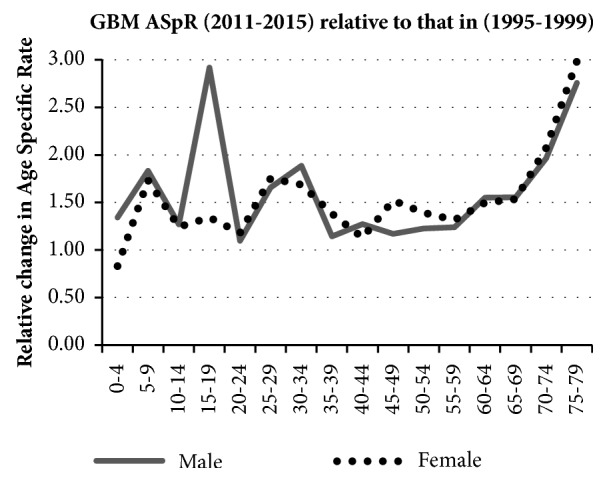
Relative change in GBM age–specific incidence rates (ASpR) averaged over two five-year periods 1995-1999 and 2011-2015 in 5-year age bands and gender.

Figure 4 shows the relative increase in age-specific GBM incidence between the averaged periods (1995–1999) and (2011–2015) for 5–year age–groups. This 1.5-fold change is remarkably similar across the age–groups, suggesting a universal factor.

**Figure 5:**
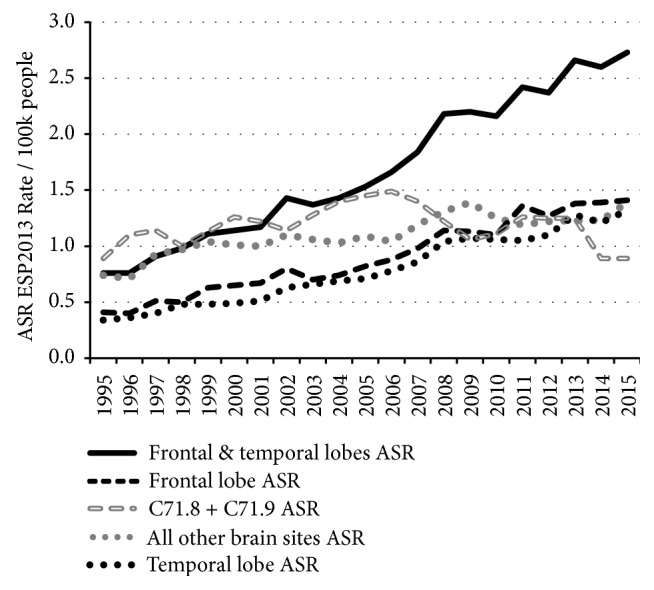
Frontal and temporal lobe GBM age–standardised incidence rates by tumour site and year (data table in the SI as [S6]).

Figure 5 shows ASR GBM rates for frontal lobe, temporal lobe, unspecified & overlapping (C71.8 & C71.9) and ‘all other brain regions'. Most of the rise is in the frontal and temporal lobes, and most of the cases are in people over 55 years of age, with a highly statistically significant overall AAPC of 7.6% (see Table 4). There was an extra rise in frontal and temporal GBM incidence between 2006 and 2008, which coincided with a slight reduction in the GBM ASR in overlapping and unspecified regions and may be due to improved imaging.

## 4. Discussion

Using sufficiently high–quality data, we present a clearer picture of the changing pattern in incidence of brain tumour types than any previously published. We report a sustained and highly statistically significant ASR rise in GBM across all ages and throughout the 21 years (1995–2015), which is of importance both for clinical resources and brain tumour aetiology.

Dobes et al. (2011) [13] reported a significant increase in malignant tumour incidence from 2000 to 2008 in the ≥65–year age group. In a second article they noted an increasing incidence of GBM (APC, 3.0; 95% CI, 0.5–5.6) in patients in the same age group, especially in temporal and frontal lobes [14]. De Vocht et al. (2011) [15] reported a rise in temporal lobe tumour incidence in ONS data, but dismissed its significance. In a 2016 paper he claimed no increase in GBM incidence, but later published a major correction to the paper that shows an increase [16].

Zada et al. (2012) [17] using USA SEER data for 1992–2006 reported a rising trend in frontal and temporal lobe tumours, the majority of which were GBM, with a decreased incidence of tumours across all other anatomical sub–sites. Ho et al. (2014) [18] reported a 2.2–fold increase in glioblastoma incidence in the Netherlands over the period 1989–2010 (APC 3.1, p<0.001).

There were no material classification changes over the analysis period that might explain our findings [19], though multidisciplinary team working was strengthened (2005 onwards) and better imaging has resulted in improved diagnosis along with a more complete registration of brain tumours in the elderly. We analysed our data in 5-year age group categories to look for evidence of improved diagnosis; the data do suggest diagnosis and registration have improved in people aged over 70. However, at earlier ages the incidence rate of ‘all' glioma (and all C71) registrations have remained almost constant, whereas the rates for lower–grade tumours fell until about 2006 and have since remained fairly static as the rate for GBM has risen steadily.

Most GBM cases seem to originate without any known genetic predisposition. GBMs from promoted lower–grade gliomas usually have different molecular genetic markers from* de novo* GBMs [20]. The 2016 revision of the WHO classification of CNS tumours [3, 4] highlights the need for recording molecular genetic markers and divides glioblastomas into two main groups. The IDH–wildtype mostly corresponds to clinically defined primary or* de novo* glioblastoma and accounts for about 90% of cases. The remaining 10% are IDH–mutant cases, which usually arise in younger patients and mostly correspond to secondary or promoted lower–grade diffuse glioma [11, 21]. Figures 3(a) and 3(b) support the conclusion of Ohgaki and Kleihues [11] that promoted (secondary) tumours mainly occur in younger people and that* de novo* GBMs dominate in the over-54 age group. It is important that this pattern is monitored using modern genetic techniques.

GBM tumours are almost always fatal and are not likely to have been undiagnosed in the time-frame of our data. It is possible that some elderly cases were not fully classified, but then they should have been recorded as ICD10–D43. However, as D43 rates have remained very constant over this time period (see Figure 1), this is unlikely to have been a significant confounder.

### 4.1. Possible Causal Factors

We cite examples of some possible causal factors that have been discussed in the literature that could contribute changes in GBM incidence. In an important 2014 “state of science” review of glioma epidemiology, Ostrom et al. [22] list and discuss a number of potential factors that have been associated with glioma incidence, some of which we list below.

Ionising radiation, especially from X-rays used in CT scans, has the most supportive evidence as a causal factor. Due to the easy availability of CT imaging and relative lack and higher cost of MRI imaging in UK NHS hospitals, CT scans are often used, especially for initial investigations. Their use over the period 1995-2013 is shown in the Supplementary File [S6]. Given the time-frame of the trend that we have identified, we suggest that CT imaging X-ray exposures should be further investigated for both the promotion and initiation of the rising incidence of GBM tumours that we have identified.

Preston et al. (2007) [23] concluded that radiation–associated cancer persists throughout life regardless of age at exposure and that glioma incidence shows a statistically significant dose response. Our oldest age group also experienced atmospheric atomic bomb testing fallout and some association with ingested and inhaled radionuclides should not be dismissed as a possible factor. England was in one of the highest exposed regions for atmospheric testing fallout as determined by the United Nations Scientific Committee on the Effects of Atomic Radiation, UNSCEAR 2000 Report [24]. Further information is given in Supplementary File S7. If only some of the population were susceptible and received a significant dose, any resulting extra cancers would show up in the ONS data.

The European Study of Cohorts for Air Pollution Effects by Andersen et al. (2017) [25] found suggestive evidence of an association between traffic-related air pollution and malignant brain tumours.

There is increasing evidence literature that many cancers including glioma have a metabolic driver due to mitochondrial dysfunction resulting in downstream genetic changes in the nucleus [26–28].

The International Agency for Research on Cancer (IARC) judged both power–frequency ELF (2002) [29] and radio–frequency RF (2011) [30] electromagnetic fields as Group 2B ‘possible human carcinogens'. Villeneuve et al. (2002) [31] concluded that occupational (ELF) magnetic field exposure increases the risk of GBM with an OR = 5.36 (95% CI: 1.2 – 24.8). Hardell and Carlberg (2015) [32] have reported an increase in high–grade glioma associated with mobile phone use. The multi-country Interphone study [33] collected data from 2000 to 2003 and included few people over 55 years of age and would have been unable to resolve any association involving older–aged people. Volkow et al. (2011) [34] found that, in healthy participants and compared with no exposure, a 50-minute cell phone exposure produced a statistically significant increase in brain glucose metabolism in the orbitofrontal cortex and temporal pole regions closest to the handset.

## 5. Conclusions

1. We show a linear, large and highly statistically significant increase in primary GBM tumours over 21 years from 1995–2015, especially in frontal and temporal lobes of the brain. This has aetiological and resource implications.
2. Although most of the cases are in the group over 54 years of age, the age–standardised AAPC rise is strongly statistically significant in all our three main analysis age groups.
3. The rise in age–standardised incidence cannot be fully accounted for by improved diagnosis, as it affects specific areas of the brain and just one type of brain tumour that is generally fatal. We suggest that widespread environmental or lifestyle factors may be responsible, although these results do not provide additional evidence for the role of any particular risk factor.
4. Our results highlight an urgent need for funding more research into the initiation and promotion of GBM tumours. This should include the use of CT imaging for diagnosis and also modern lifestyle factors that may affect tumour metabolism.

## Figures and Tables

**Table 1:** ONS WHO ICD10 brain tumour data for England.

		**1995**	**2015**
**C71**	Malignant primary neoplasm of brain	**cases**	**cases**
C71.0	Cerebrum except lobes & ventricles	154	213
C71.1	Frontal lobe	533	1231
C71.2	Temporal lobe	334	994
C71.3	Parietal lobe	506	587
C71.4	Occipital lobe	95	162
C71.5	Cerebral ventricle	31	47
C71.6	Cerebellum	138	143
C71.7	Brain stem	72	99
C71.8	Overlapping lesion of brain	262	208
C71.9	Brain, unspecified site	1286	770

**C71**	**All topology sites**	**3411**	**4454**

D43	Uncertain behaviour (no histology data)		
		**1998**	**2015**
D43.0-43.2	Unspecified tumour details - cases	361	383

**Table 2:** **ICD-O-3 morphology codes with more than 100 cases between 1995-2015 inclusive.** (A full listing of all the morphology codes and cases is present in the Supplementary file).

Morphology	Grade	All cases	Group	Sub-group	WHO/IARC summary description
80003	1	7776	NOS		unclassified, malignant, blastoma, NOS
80013	2	250	carcinoma		carcinoma, metastatic, NOS
80103	1	536	carcinoma		epithelial tumour, carcinoma, malignant
80106	2	281	carcinoma		carcinoma, metastatic, NOS
89633	2	131	sarcoma		rhabdoid sarcoma
90643	2	106			germ cell neoplasia
93803	2	11269	glioma	NOS	glioma, malignant, NOS, not neoplastic
93813	3	187	glioma	astrocytic	gliomatosis cerebri
93823	2	1298	glioma	astrocytic	mixed glioma / oligoastrocytoma
93913	2	1034	glioma	ependymal	ependymoma
93923	3	313	glioma	ependymal	anaplastic ependymoma
94003	2	7807	glioma	astrocytic	astrocytoma, NOS, diffuse
94013	3	2832	glioma	astrocytic	anaplastic astrocytoma (high grade)
94113	2	331	glioma	astrocytic	germistocytic astrocytoma, diffuse
94203	2	420	glioma	astrocytic	fibrillary astrocytoma, diffuse
94213	1	2125	glioma	astrocytic	pilocytic astrocytoma
94243	2	106	glioma	astrocytic	pleomorphic xantoastrocytoma
94403	4	37046	glioma	GBM-IV	glioblastoma multiforme
94413	4	263	glioma	GBM-IV	giant cell glioblastoma
94423	4	477	glioma	GBM-IV	gliosarcoma
94503	2	2671	glioma	oligodendrial	oligodendroglioma
94513	3	1339	glioma	oligodendrial	anaplastic oligodendroglioma
94703	4	1178	glioma	embryonal	medulloblastoma
94713	4	106	glioma	embryonal	desmoplastic medulloblastoma
94733	4	472	glioma	embryonal	primitive neuroectodermal tumour

**Table 3:** ICD10-C71 and (D43.0 + D43.2) cases and age-standardised (ESP-2013) incidence rates.

Type ->	GBM	astro-c non_GBM	glioma 93803	Other glioma	other C71	D43.0 +D43.2	GBM	astro-c non_GBM	glioma 93803	Other glioma	other C71	all C71	D43.0 +D43.2
**Year**	Case numbers						Age-standardised (ESP-2013) incidence rates						

**1995**	983	925	736	339	428	n/a	2.39	2.04	1.79	0.69	1.06	7.97	n/a
**1996**	1064	852	714	313	455	n/a	2.57	1.87	1.73	0.66	1.10	7.93	n/a
**1997**	1232	820	725	353	483	n/a	2.98	1.80	1.74	0.73	1.16	8.41	n/a
**1998**	1238	854	663	353	435	361	2.95	1.85	1.58	0.72	1.05	8.15	0.86
**1999**	1384	755	560	330	522	447	3.45	1.61	1.41	0.66	1.01	8.14	1.06
**2000**	1449	770	528	404	631	445	3.41	1.64	1.24	0.83	1.49	8.61	1.04
**2001**	1449	761	554	403	479	459	3.39	1.59	1.29	0.82	1.14	8.23	1.06
**2002**	1576	644	542	443	493	431	3.67	1.33	1.25	0.91	1.14	8.30	0.98
**2003**	1605	630	484	408	446	443	3.71	1.30	1.11	0.82	1.04	7.98	1.01
**2004**	1686	573	505	428	435	441	3.86	1.17	1.15	0.86	1.01	8.05	1.00
**2005**	1802	559	484	447	480	492	4.07	1.12	1.10	0.89	1.09	8.27	1.08
**2006**	1866	546	462	425	499	440	4.19	1.10	1.03	0.83	1.12	8.27	0.97
**2007**	1998	525	436	496	455	457	4.43	1.03	0.96	0.98	1.00	8.40	0.99
**2008**	2152	569	488	443	428	486	4.71	1.11	1.06	0.86	0.94	8.68	1.03
**2009**	2152	509	538	450	500	421	4.64	0.99	1.17	0.86	1.07	8.73	0.88
**2010**	2111	551	470	483	492	485	4.51	1.05	1.00	0.91	1.05	8.52	0.99
**2011**	2314	518	462	475	393	467	4.86	0.98	0.97	0.90	0.84	8.55	0.96
**2012**	2330	524	459	418	433	437	4.84	0.98	0.95	0.79	0.88	8.44	0.89
**2013**	2518	591	472	425	424	450	5.15	1.11	0.97	0.79	0.87	8.89	0.88
**2014**	2349	621	462	464	371	463	4.73	1.15	0.91	0.86	0.73	8.38	0.89
**2015**	2531	602	525	450	346	383	5.02	1.12	1.02	0.82	0.65	8.63	0.73

**Table 4:** Age standardised incidence rates to ESP-2013 (/100k people).

**Year **	**GBM all brain sites**				all ages	all ages	**GBM frontal and temporal lobes**				all ages	all ages
**age**-**>**	< 30	30-54	55+	all ages	M	F	< 30	30-54	55+	all ages	M	F
**AAPC**	**2.6**	**1.7**	**4.1**	**3.6**	**3.5**	**3.7**	**5.6**	**4.7**	**7.6**	**6.9**	**6.8**	**6.9**
**CI**	1.5 3.9	1.2 2.2	3.5 4.7	3.1 4.1	2.9 4.1	3.2 4.1	4.0 7.3	3.9 5.5	7.0 8.2	6.3 7.4	6.2 7.4	6.4 7.5
**p**	0.0002	<0.0001	<0.0001	<0.0001	<0.0001	<0.0001	<0.0001	<0.0001	<0.0001	<0.0001	<0.0001	<0.0001

**1995**	0.13	1.87	5.33	2.39	2.99	1.85	0.03	0.64	1.64	0.76	0.90	0.62
**1996**	0.16	1.95	5.77	2.57	3.22	1.98	0.04	0.66	1.64	0.76	0.98	0.57
**1997**	0.19	1.87	7.1	2.98	3.87	2.21	0.06	0.66	2.08	0.91	1.17	0.68
**1998**	0.22	2.08	6.73	2.95	3.74	2.23	0.06	0.78	2.16	0.98	1.31	0.67
**1999**	0.20	2.34	7.51	3.28	4.28	2.35	0.06	0.99	2.34	1.11	1.42	0.82
**2000**	0.24	2.34	7.87	3.41	4.39	2.52	0.08	0.94	2.45	1.14	1.50	0.81
**2001**	0.25	2.14	8.04	3.39	4.30	2.58	0.11	0.89	2.59	1.17	1.50	0.89
**2002**	0.15	2.39	8.73	3.67	4.76	2.72	0.06	1.03	3.29	1.43	1.92	0.99
**2003**	0.24	2.22	8.95	3.71	4.84	2.69	0.07	0.89	3.23	1.37	1.81	0.97
**2004**	0.19	2.33	9.35	3.86	5.00	2.87	0.07	1.06	3.25	1.43	1.82	1.07
**2005**	0.23	2.55	9.74	4.07	5.39	2.88	0.09	0.94	3.66	1.53	2.00	1.1
**2006**	0.25	2.41	10.25	4.19	5.35	3.16	0.10	1.06	3.95	1.66	2.13	1.22
**2007**	0.26	2.51	10.88	4.43	5.68	3.34	0.09	1.07	4.50	1.84	2.35	1.38
**2008**	0.25	2.82	11.41	4.71	5.91	3.63	0.12	1.51	5.05	2.18	2.73	1.66
**2009**	0.24	2.66	11.39	4.64	5.88	3.53	0.08	1.39	5.30	2.20	2.79	1.66
**2010**	0.23	2.52	11.14	4.51	5.75	3.41	0.10	1.39	5.15	2.16	2.82	1.55
**2011**	0.26	2.62	12.1	4.86	6.04	3.82	0.11	1.56	5.76	2.42	3.05	1.84
**2012**	0.27	2.57	12.07	4.84	6.22	3.61	0.10	1.49	5.71	2.37	3.10	1.72
**2013**	0.30	2.60	12.97	5.15	6.64	3.80	0.11	1.49	6.59	2.66	3.47	1.91
**2014**	0.22	2.44	11.93	4.73	6.02	3.59	0.14	1.45	6.43	2.60	3.27	2
**2015**	0.32	2.83	12.28	5.02	6.26	3.91	0.15	1.65	6.60	2.73	3.33	2.18

## Data Availability

The data were obtained from the UK Office for National Statistics (ONS), who are the legal owners of the data. Some data are publicly available in the ONS annual MB1 data series, which are freely downloadable from the ONS website, but this article uses the latest updated data, plus ICD–O–3 morphology codes, extracted under personal researcher contract from the ONS database in July 2017. ONS Data Guardian approval was required for the supply, control and use of the data. A nominal charge is made by the ONS for such data extraction. We are not permitted to supply the raw ONS extracted data to anyone else. Other researchers can obtain the latest data directly from the ONS in a similar manner. The authors provide some extra tables and figures in the Supplementary File downloadable from the journal website.

## Supplementary Materials

Supplementary Materials S1. Table of data morphology coding and the case numbers used in the study. S2. GBM case numbers and age-specific incidence rate data used in the study. S3. Sample STATA data and DO script. S4. Data table for Figure 1. S5. Data table for Figure 5. S6. CT and MRI use in the UK NHS. S7. Some notes on atomic bomb testing and other nuclear fallout in England.
