# Supplementary material for: Brain Tumours: Rise in Glioblastoma Multiforme Incidence in England 1995–2015 Suggests an Adverse Environmental or Lifestyle Factor
Source: J Environ Public Health. 2018 Jun 24;2018:7910754. doi: 10.1155/2018/7910754 (PMC6035820; doi:10.1155/2018/7910754)
Supplement: Supplementary Materials — S1. Table of data morphology coding and the case numbers used in the study. S2. GBM case numbers and age-specific incidence rate data used in the study. S3. Sample STATA data and DO script. S4. Data table for Figure 1. S5. Data table for Figure 5. S6. CT and MRI use in the UK NHS. S7. Some notes on atomic bomb testing and other nuclear fallout in England. [file 7910754.f1.pdf]

Rise in Glioblastoma Multiforme incidence in England 1995–2015 suggests an adverse environmental or lifestyle factor

Alasdair Philips, Denis L Henshaw, Graham Lamburn, Michael J O'Carroll

Supplementary Information

CONTENTS

S1. Table of data morphology coding and the case numbers used in the study

S2. GBM case numbers and age-specific incidence rate data used in the study

S3. Sample STATA data and DO script

S4. Data-table for Figure 1

S5. Data-table for Figure 5

S6. CT and MRI use in the UK NHS

S7. Some notes on atomic bomb testing and other nuclear fallout in England

Data Sharing

The cancer incidence data used the main paper and in this Supplement were obtained from the UK Office for National Statistics (ONS) who are the legal owners of the data. Some data are publicly available in the ONS annual MB1 data series which are freely downloadable from the ONS website, but the main paper uses updated data, plus ICD–O–3 morphology codes, extracted under personal researcher contract from the ONS database in September 2017. ONS Data Guardian approval was required for the supply, control and use of the data. A nominal charge is made by the ONS for such data extraction. We are not permitted to supply the raw ONS extracted data to anyone else. Other *bone–fide* researchers can obtain the latest data directly from the ONS in a similar manner.

S1/. <u>All ICD10-O-codes in the data-set with descriptions and total case numbers</u>

There are 103 ICD-O-3 codes used in the full dataset. Some detailed descriptions and categories may be debated slightly by clinicians, but this would make little difference to the overall results of our analyses.

Morphology	group	sub	subsub	Grade	cases 1995-2015	Description
**unspecified:**					a	Only 1 to 5 cases over the period 1995-29015 inclusive
80001				**1**	a	neoplasm uncertain if malignant
80003				**2**	**7776**	unclassified, malignant, NOS
80006				**2**	**18**	neoplasm, metastaic (secondary)
80009				**2**	**19**	neoplasm, malignant, uncertain whether metastatic
80013				**2**	**250**	carcinoma, metastatic, NOS
80023				**2**	a	malignant tumour, small cell type
80033				**2**	a	malignant tumour, giant cell type
80043				**2**	a	malignant tumour, spindle cell or fusiform cell type
**other:**						
80103				**2**	**536**	epithelial tumour, carcinoma, malignant
80106				**2**	**281**	carcinoma, metastatic, NOS
80109				**2**	**28**	carcinomatosis
80426				**2**	a	oat cell carcinoma
80463				**2**	a	carcinoma
80706				**2**	a	squamous cell carcinoma
81403				**2**	a	adenocarcinoma NOS
81406				**3**	**26**	adenocarcinoma,, metastatic, NOS
82463				**2**	**15**	neuroendocrine carcinoma
82466				**2**	a	? carcinoma
82469				**2**	a	? carcinoma
82606				**2**	a	papillary adenocarcinoma
83106				**2**	a	clear cell adenocarcinoma
84013				**2**	a	apocrine adenocarcinoma
84816				**2**	a	mucin producing adenocarcinma
85103				**2**	a	medullary carinoma NOS
87203				**2**	**9**	malignant melanoma NOS
87206				**2**	a	malignant melanoma
88003				**2**	**24**	sarcoma NOS
88013				**2**	**8**	spindle cell sarcoma
88023				**2**	a	giant cell sarcoma
88033				**2**	a	small cell or round cell sarcoma
88103				**2**	a	fibrosarcoma
88113				**2**	a	fibromyxosarcoma
88303				**2**	a	fibrous malignant histiocytoma
88503				**2**	a	liposarcoma NOS
88613				**2**	a	angliolipoma
88903				**2**	a	leiomyosarcoma
89003				**2**	a	rhabdomyosarcoma
89103				**2**	a	embryonal rhabdomyosarcoma
89203				**2**	a	alveolar rhabdomyosarcoma
89633				**2**	**131**	rhabdoid sarcoma
90603				**2**	a	dysgerminoma
90643				**2**	**106**	germ cell neoplasia
90801				**1**	a	teratoma NOS
90803				**2**	**7**	teratoma, malignant, NOS
90853				**2**	a	mixed germ cell tumour
91003				**2**	a	choriocarcinoma
91203				**2**	a	hermangiosarcoma
91313				**2**	a	capillary herangioma
91333				**2**	a	epithelioid hermangioendothelioma
91503				**2**	**56**	hermangiopericytoma
91613				**2**	**43**	hermangioblastoma / angioblastoma
91703				**2**	a	lymphangiosarcoma
92203				**2**	a	
92313				**2**	a	myxoid chondrosarcoma
92403				**2**	a	mesenchymal chondrosarcoma
93643				**2**	**42**	peripheral neuroectodermal tumour
93703				**2**	**50**	chordoma
95303				**3**	**58**	anaplastic meningioma
95313				**3**	a	meningiotheliomatous meningioma
95603				**3**	a	neurilemoma, malignant
95613				**3**	a	triton tumour, malignant / malignant schwannoma

X<br>Morphology	group	sub	subsub	Grade	cases 1995-2015	Description
**neuroepithelial:**		**938 to 948 incl. are WHO classed as gliomas**				
					a	Only 1 to 5 cases over the period 1995-2015 inclusive
93803	**GLIO**		**GL2**	**2**	**11269**	glioma, malignant, NOS, not neoplastic
93813	**GLIO**	**AST**	**AST3**	**3**	**187**	gliomatosis cerebri
93823	**GLIO**	**AST**	**OGL2**	**2**	**1298**	mixed glioma / oglioastrocytoma
93833	**GLIO**	**AST**	**EPN**	**1**	**8**	subependymal glioma / astrocytoma
93843	**GLIO**	**AST**	**EPN**	**1**	a	subependymal giant cell astrocytoma
93903	**GLIO**		**EPN**	**3**	**77**	choroid plexus carcinoma
93913	**GLIO**		**EPN**	**2**	**1034**	ependymoma
93923	**GLIO**		**EPN**	**3**	**313**	anaplastic ependymoma
93933	**GLIO**		**EPN**	**3**	a	papillary ependymoma
93943	**GLIO**		**EPN**	**1**	a	myxopapillary ependymoma
94003	**GLIO**	**AST**	**AST2**	**2**	**7807**	astrocytoma, NOS, diffuse
94013	**GLIO**	**AST**	**AST3**	**3**	**2832**	anaplastic astrocytoma (high grade)
94103	**GLIO**	**AST**	**AST3**	**3**	**29**	anaplastic astrocytoma
94113	**GLIO**	**AST**	**AST2**	**2**	**331**	gemistocytic astrocytoma, diffuse
94203	**GLIO**	**AST**	**AST2**	**2**	**420**	fibrillary astrocytoma, diffuse
94213	**GLIO**	**AST**	**AST1**	**1**	**2125**	pilocytic astrocytoma
94243	**GLIO**	**AST**	**AST2**	**2**	**106**	pleomorphic xantoastrocytoma
94303	**GLIO**	**AST**	**AST1**	**1**	**33**	astroblastoma
94403	**GLIO**	**AST**	**GBM**	**4**	**37046**	glioblastoma multiforme
94406	**GLIO**	**AST**	**GBM**	**4**	a	glioblastoma G4 NOS
94413	**GLIO**	**AST**	**GBM**	**4**	**263**	giant cell glioblastoma
94423	**GLIO**	**AST**	**GBM**	**4**	**477**	gliosarcoma
94433	**GLIO**	**AST**		**4**	a	polar spongioblastoma
94503	**GLIO**		**OGD2**	**2**	**2671**	oligodendroglioma
94513	**GLIO**		**OGD3**	**3**	**1339**	anaplastic oligodendroglioma
94603	**GLIO**			**2**	**54**	oligodendroblastoma
94703	**Glio?**		**EMB**	**4**	**1178**	medulloblastoma
94713	**Glio?**		**EMB**	**4**	**106**	desmoplastic medulloblastoma
94723	**Glio?**		**EMB**	**4**	**16**	medullomyoblastoma
94733	**Glio?**		**EMB**	**4**	**472**	primitive neuroectodermal tumour
94803	**GLIO**		*obsolete*	**2**	a	cerebellar sarcoma
94813	**GLIO**		*obsolete*	**2**	a	monstrocellular sarcoma
94903			**EMB**	**2**	a	ganglioneuroblastoma
95003			**EMB**	**4**	**10**	neuroblastoma NOS
95013			**EMB**	**3**	a	medulloepithelioma NOS
95033			**EMB**	**3**	a	neuroepithelioma NOS
95053				**3**	**43**	anaplastic ganglioglioma
95063				**3**	a	neurocytoma

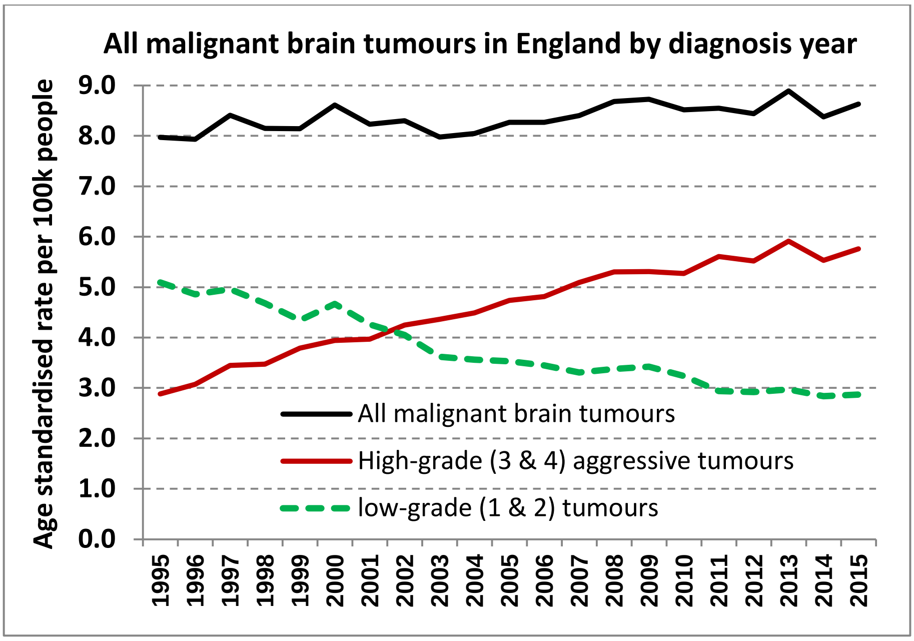

S2/. Case numbers and age-specific incidence rate data tables for GBM used in the study

**Raw case numbers for GBM for all genders and all regions of the brain** (Note: a = 1 to 5 cases)																			
**Age Group**	0-4	5-9	10-14	15-19	20-24	25-29	30-34	35-39	40-44	45-49	50-54	55-59	60-64	65-69	70-74	75-79	80-84	85+	**Totals**
**1995**	a	a	a	a	a	8	12	23	57	79	114	144	167	154	128	55	22	a	983
**1996**	6	a	a	a	6	14	19	21	51	92	123	150	153	186	149	66	19	a	1064
**1997**	a	a	a	a	8	14	25	25	55	74	124	186	193	225	159	95	20	14	1232
**1998**	a	a	a	a	13	15	25	46	43	86	144	153	198	202	170	100	21	9	1238
**1999**	0	6	a	6	9	11	17	45	62	116	146	186	223	248	166	108	23	7	1384
**2000**	a	6	a	a	14	14	31	42	59	100	160	186	215	235	208	133	24	12	1449
**2001**	a	a	a	9	a	23	20	36	62	90	151	203	222	252	187	121	44	15	1449
**2002**	a	a	a	a	8	8	28	44	56	103	166	217	250	274	195	141	55	19	1576
**2003**	a	7	a	a	7	19	25	35	49	96	160	222	252	268	239	141	58	16	1605
**2004**	a	9	6	a	7	7	23	51	58	104	151	225	252	286	253	154	72	21	1686
**2005**	a	6	8	a	6	14	35	42	87	103	161	259	247	305	273	153	70	23	1802
**2006**	a	a	a	a	11	20	27	33	78	97	169	272	307	294	248	194	65	35	1866
**2007**	a	6	a	13	10	13	25	47	76	122	159	268	345	305	293	192	82	34	1998
**2008**	7	a	a	a	14	17	35	42	76	136	196	264	379	315	322	189	99	50	2152
**2009**	a	a	a	11	12	13	22	50	67	133	192	255	384	351	273	215	99	63	2152
**2010**	6	a	a	7	10	15	19	37	67	115	206	225	363	336	299	227	111	60	2111
**2011**	a	8	7	7	11	18	28	33	84	132	194	273	391	379	288	246	144	70	2314
**2012**	a	a	a	10	12	21	27	43	62	142	193	241	371	379	330	256	140	92	2330
**2013**	a	7	a	9	15	20	40	35	79	132	192	258	397	461	361	269	145	89	2518
**2014**	a	a	a	9	8	18	36	37	77	116	185	246	324	421	352	264	149	98	2349
**2015**	a	7	6	10	9	29	33	40	83	161	212	259	339	416	356	306	171	90	2531

Age specific rates (non-adjusted incidence rates per 100,000 people) for GBM for all genders and all regions of the brain																			
**Age Group**	**0-4**	**5-9**	**10-14**	**15-19**	**20-24**	**25-29**	**30-34**	**35-39**	**40-44**	**45-49**	**50-54**	**55-59**	**60-64**	**65-69**	**70-74**	**75-79**	**80-84**	**85+**	**Totals**
**1995**	0.13	0.06	0.10	0.14	0.12	0.21	0.31	0.68	1.82	2.31	4.10	5.74	7.20	6.97	6.19	3.77	1.99	0.35	2.03
**1996**	0.19	0.03	0.10	0.07	0.19	0.37	0.49	0.60	1.62	2.68	4.24	6.02	6.62	8.44	7.40	4.30	1.73	0.34	2.19
**1997**	0.13	0.12	0.13	0.10	0.27	0.38	0.63	0.70	1.73	2.26	3.95	7.44	8.31	10.26	8.04	5.92	1.87	1.58	2.53
**1998**	0.07	0.09	0.13	0.13	0.45	0.42	0.64	1.25	1.33	2.71	4.39	6.00	8.41	9.25	8.69	5.95	2.06	0.98	2.54
**1999**	0.00	0.19	0.16	0.20	0.31	0.31	0.43	1.19	1.87	3.70	4.37	7.09	9.33	11.45	8.53	6.26	2.33	0.75	2.82
**2000**	0.10	0.19	0.09	0.13	0.48	0.41	0.80	1.09	1.74	3.21	4.74	6.86	8.92	10.88	10.68	7.92	2.29	1.26	2.94
**2001**	0.14	0.03	0.15	0.30	0.13	0.69	0.52	0.92	1.78	2.88	4.49	7.17	9.27	11.67	9.58	7.36	3.93	1.56	2.93
**2002**	0.07	0.16	0.12	0.03	0.26	0.25	0.74	1.11	1.56	3.26	5.15	7.09	10.43	12.60	9.99	8.69	4.68	1.99	3.17
**2003**	0.07	0.23	0.15	0.13	0.22	0.60	0.67	0.88	1.33	2.98	5.13	6.96	10.31	12.17	12.26	8.75	4.71	1.71	3.21
**2004**	0.10	0.29	0.19	0.12	0.22	0.22	0.63	1.29	1.54	3.16	4.91	6.94	10.03	12.81	13.05	9.56	5.73	2.24	3.36
**2005**	0.17	0.20	0.25	0.15	0.18	0.43	0.98	1.07	2.25	3.06	5.27	7.92	9.54	13.56	14.09	9.44	5.67	2.33	3.56
**2006**	0.10	0.10	0.16	0.15	0.33	0.60	0.78	0.85	1.99	2.80	5.51	8.31	11.36	13.21	12.79	11.88	5.34	3.37	3.66
**2007**	0.13	0.20	0.13	0.39	0.29	0.38	0.74	1.22	1.92	3.43	5.12	8.59	11.80	13.67	14.98	11.67	6.77	3.14	3.89
**2008**	0.22	0.14	0.16	0.06	0.40	0.48	1.05	1.11	1.92	3.73	6.18	8.71	12.40	13.80	16.16	11.44	8.17	4.50	4.15
**2009**	0.12	0.14	0.13	0.33	0.35	0.36	0.65	1.35	1.70	3.55	5.92	8.50	12.34	14.88	13.46	12.99	8.10	5.55	4.12
**2010**	0.18	0.17	0.10	0.21	0.28	0.41	0.55	1.02	1.72	3.00	6.19	7.54	11.52	13.76	14.60	13.64	8.93	5.15	4.01
**2011**	0.03	0.27	0.23	0.21	0.31	0.49	0.79	0.94	2.16	3.39	5.67	9.09	12.36	14.82	14.16	14.66	11.38	5.87	4.36
**2012**	0.15	0.13	0.07	0.30	0.33	0.57	0.75	1.26	1.61	3.62	5.48	7.89	12.31	13.57	16.08	15.02	10.87	7.54	4.36
**2013**	0.12	0.22	0.17	0.28	0.42	0.54	1.09	1.04	2.08	3.36	5.31	8.28	13.50	15.80	17.14	15.43	11.15	7.19	4.67
**2014**	0.12	0.06	0.10	0.28	0.22	0.48	0.97	1.09	2.08	2.96	4.98	7.72	11.12	14.15	16.09	14.79	11.34	7.68	4.32
**2015**	0.12	0.21	0.20	0.31	0.25	0.77	0.89	1.15	2.28	4.14	5.56	7.90	11.67	13.79	15.69	16.96	12.89	6.95	4.62

S3/. Sample simple STATA ® dataset and DO script

Statistical analysis was carried out using STATA ® v12.1 (StataCorp LP, Texas. USA)

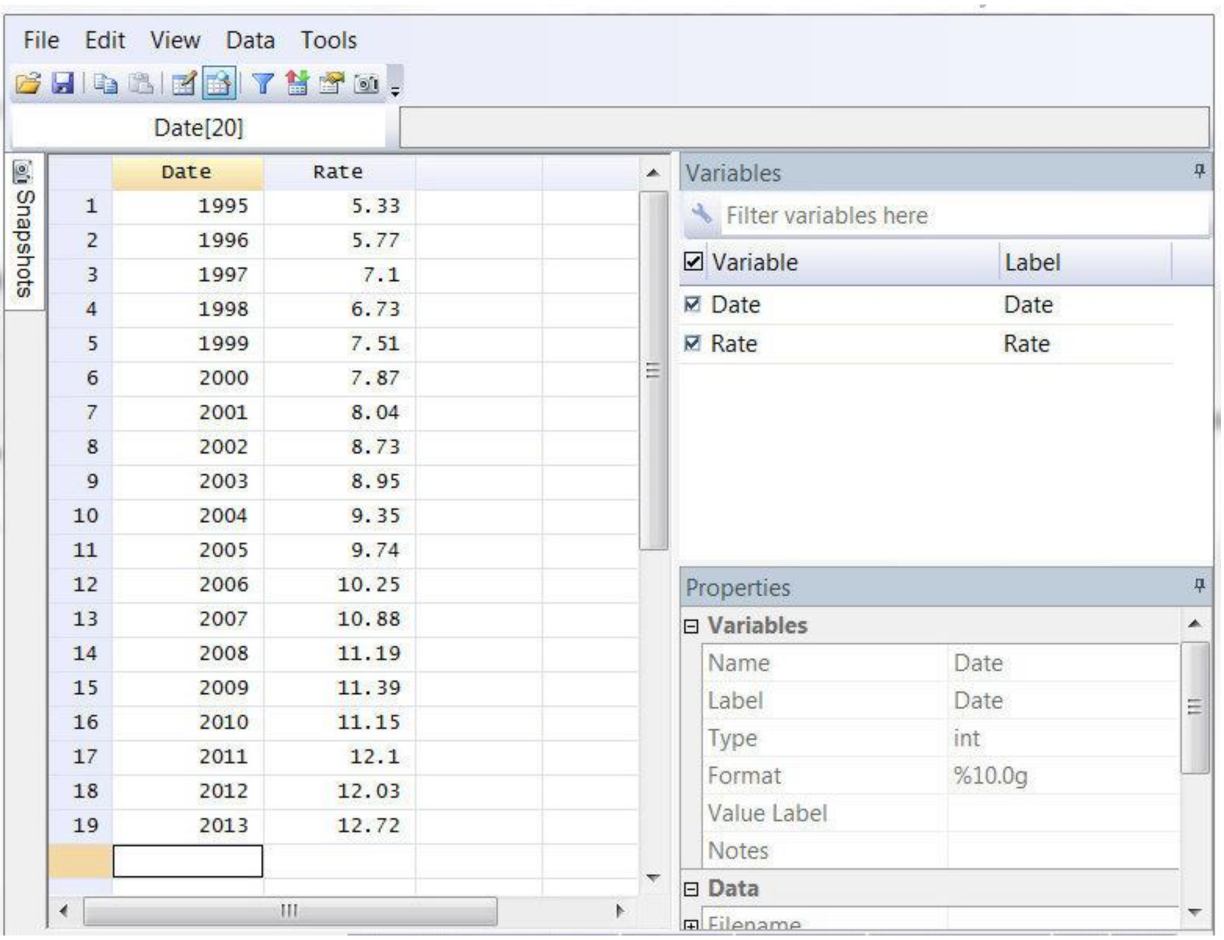


```
. use "c:\ons\015stata\16aapc.dta", clear
. *This is the local directory where the file is located
. generate rate_log=log(rate)
. regress rate_log year
      Source |       SS       df       MS              Number of obs =      19
-------------+------------------------------           F(  1,    17) =   53.31
       Model |  .188982226     1  .188982226           Prob > F      =  0.0000
    Residual |  .060260949    17  .003544762           R-squared     =  0.7582
-------------+------------------------------           Adj R-squared =  0.7440
       Total |  .249243175    18  .013846843           Root MSE      =  .05954

------------------------------------------------------------------------------
    rate_log |      Coef.   Std. Err.      t    P>|t|     [95% Conf. Interval]
-------------+----------------------------------------------------------------
        year |   .0182085   .0024938     7.30   0.000     .0129471    .0234698
       _cons |  -35.64104    4.99753    -7.13   0.000    -46.18491   -25.09717
------------------------------------------------------------------------------
. local t = _b[year]/_se[year]
. di 2*ttail(e(df_r),abs(`t'))
1.237e-06
. *p value to more decimal places

. generate exp_year_rate=(exp(_b[year])-1)*100
. generate exp_year_rate_down=(exp(_b[year]-(invttail(e(df_r),.025)*_se[year]))> -1)*100
. generate exp_year_rate_up=(exp(_b[year]+(invttail(e(df_r),.025)*_se[year]))-1> )*100
. fsum exp_year_rate exp_year_rate_down exp_year_rate_up, stats (mean) format (> %9.4f)
      Variable             N      Mean
--------------------+------------------
      exp_year_rate |       19    1.8375
 exp_year_rate_down |       19    1.3031
   exp_year_rate_up |       19    2.3747
. *Here you find the AAPC and 95 % CI
end of do-file
```

S4/. Data-Table for Figure 1 (Values taken directly from ONS published data)

Figure 1 data table

Year	Male	Female	Combined	D43 1,2,3	Male	Female	Combined
1971	6.4	4.0	5.2				
1972	6.2	3.9	5.1				
1973	5.6	3.7	4.7				
1974	5.9	4.1	5.0				
1975	6.0	3.7	4.9				
1976	5.9	3.8	4.9				
1977	6.0	4.1	5.1				
1978	6.1	3.8	5.0				
1979	7.2	4.6	5.9				
1980	7.2	4.8	6.0				
1981	7.5	4.9	6.2				
1982	7.2	4.8	6.0				
1983	7.7	5.1	6.4				
1984	7.9	5.2	6.6				
1985	8.3	5.4	6.9				
1986	7.9	5.2	6.6				
1987	8.5	5.2	6.9				
1988	8.4	5.6	7.0				
1989	8.5	5.7	7.1				
1990	8.7	5.5	7.1				
1991	8.9	5.5	7.2				
1992	9.5	6.4	8.0				
1993	9.3	6.2	7.8				
1994	9.4	6.2	7.8				
1995	9.7	6.5	8.0				
1996	9.7	6.5	7.9				
1997	10.6	6.6	8.4				
1998	9.9	6.7	8.2	0.9			
1999	10.3	6.3	8.1	1.1			
2000	10.6	7.0	8.6	1.0			
2001	10.2	6.5	8.2	1.1	8.1	5.0	6.6
2002	10.3	6.6	8.3	1.0	7.8	5.0	6.4
2003	10.0	6.2	8.0	1.0	7.9	4.9	6.4
2004	10.2	6.2	8.1	1.0	8.2	4.8	6.5
2005	10.6	6.3	8.3	1.1	8.1	4.7	6.4
2006	10.3	6.5	8.3	1.0	8.1	5.0	6.6
2007	10.3	6.8	8.4	1.0	8.0	5.3	6.7
2008	10.6	7.0	8.7	1.0	8.2	5.0	6.6
2009	10.7	7.0	8.7	0.9	8.3	5.3	6.8
2010	10.4	6.9	8.5	1.0	8.4	5.4	6.9
2011	10.2	7.1	8.6	1.0	8.2	5.5	6.9
2012	10.6	6.5	8.4	0.9	8.8	5.6	7.2
2013	11.0	7.1	8.9	0.9	8.4	5.5	7.0
2014	10.1	6.8	8.4	0.9	8.7	5.4	7.1
2015	10.5	7.0	8.6	0.7	8.5	5.4	7.0

S5/. Data table for Figure 5

Data for Figure 6	GBM by tumour site, ASRs to ESP-2013				
Year	Frontal lobe ASR	Temporal lobe ASR	Frontal & temporal lobes ASR	Uncertain or overlapping	All other brain sites ASR
1995	0.41	0.34	0.76	0.89	0.74
1996	0.40	0.36	0.76	1.10	0.71
1997	0.51	0.40	0.91	1.14	0.93
1998	0.50	0.48	0.98	1.00	0.97
1999	0.63	0.48	1.11	1.13	1.04
2000	0.65	0.49	1.14	1.26	1.01
2001	0.67	0.51	1.17	1.22	1.00
2002	0.80	0.63	1.43	1.14	1.10
2003	0.70	0.66	1.37	1.28	1.06
2004	0.74	0.69	1.43	1.40	1.03
2005	0.82	0.71	1.53	1.45	1.09
2006	0.88	0.78	1.66	1.49	1.04
2007	0.98	0.86	1.84	1.40	1.19
2008	1.14	1.04	2.18	1.22	1.31
2009	1.13	1.07	2.20	1.06	1.39
2010	1.10	1.06	2.16	1.11	1.25
2011	1.36	1.05	2.42	1.26	1.19
2012	1.27	1.10	2.37	1.25	1.22
2013	1.38	1.28	2.66	1.25	1.23
2014	1.39	1.21	2.60	0.89	1.23
2015	1.41	1.32	2.73	0.89	1.39

S6/. CT and MRI use in the UK NHS

Source: NHS Imaging and Radio-diagnostic activity in England 2012/13 Release, August 2013
https://www.england.nhs.uk/statistics/wp-content/uploads/sites/2/2013/04/KH12-release-2012-13.pdf

Graph 2: Growth in number of Ultrasound, CT and MRI imaging and radiodiagnostic examinations or tests, England, 1995-96 to 2012-13

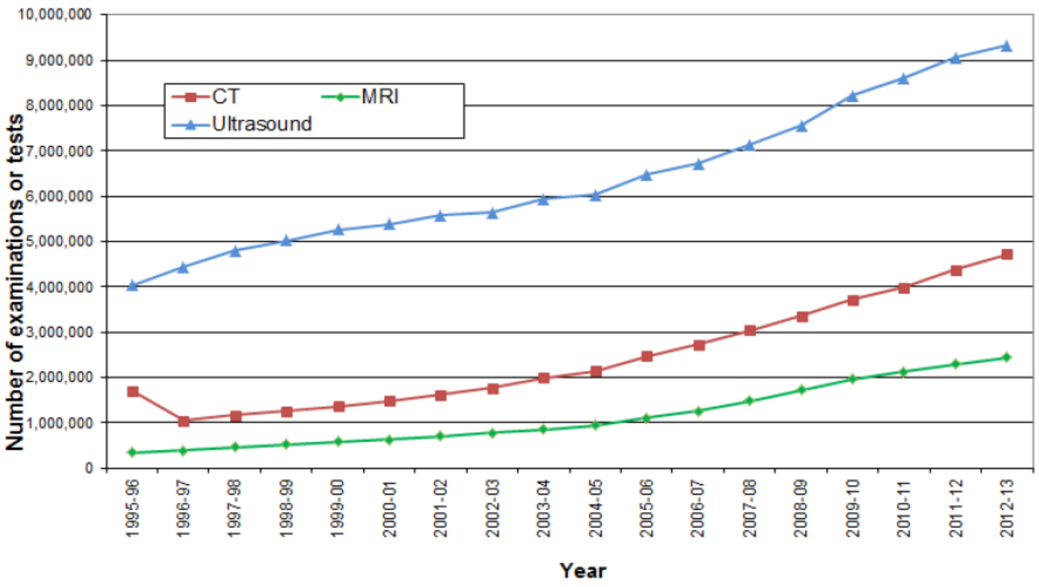

S7/. Ionising radiation in the environment from man-made sources

England lies between the latitudes of 50° and 55° North. The United Nations Scientific Committee on the Effects of Atomic Radiation UNSCEAR 2000 Report (Reference 24 in the main paper) gives the best estimates possible of overall fallout from atmospheric bomb testing. It shows that the highest levels of ^{137}Cs were around latitudes of 50° North and, although the biological half-life is generally less than six months, the fallout persisted for many years. The Caesium graph is reproduced from page 167 and the Strontium graph from page 164 of the UNCSEAR 2000 Report, with thanks.

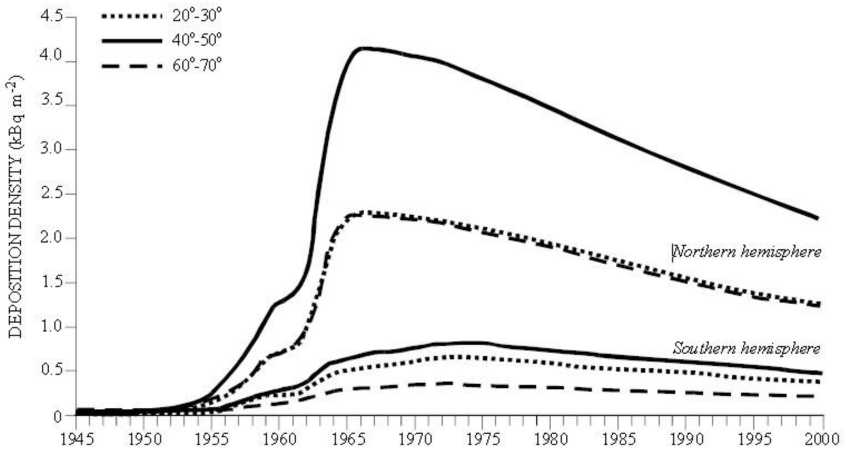

Figure VII. Caesium-137 deposition density in the northern and southern hemispheres calculated from fission production amounts with the atmospheric model.

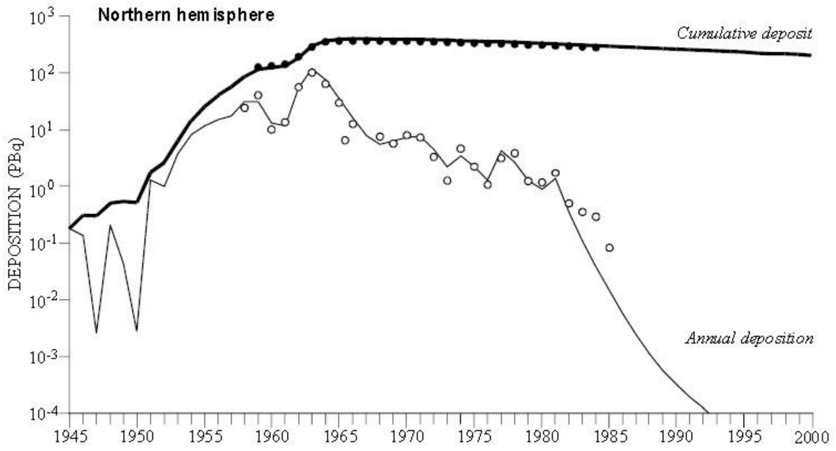

Figure V. Hemispheric depositions of ^{90}Sr determined from global network measurements (points) and from atmospheric model calculations (lines).

There were extra significant emissions of ^{137}Cs from Chernobyl that were detected in England and in a UK whole-body radiation scanning study. These are discussed and referenced in a 2010 PhD Thesis by E A Elessawi, 'Measurement of Caesium–137 in the Human Body using a Whole Body Counter'. https://orca.cf.ac.uk/55066/1/U585455.pdf

Both ^{137}Cs and ^{90}Sr are bone-seekers and are most associated with bone cancer and leukaemia.

The USA Health Physics Society has some excellent brief summaries of the effects of ^{137}Cs and ^{90}Sr.

http://hpschapters.org/northcarolina/NSDS/cesium.pdf

http://hpschapters.org/northcarolina/NSDS/strontium.pdf
